# Effect of fluoride toothpastes on surface roughness of nickel–titanium wires: an in vitro study

**DOI:** 10.2340/biid.v13.46087

**Published:** 2026-05-22

**Authors:** Karen Lisette Vega Bravo, Marjory Elizabeth Vaca Zapata, Mauricio Aguirre Balseca, Karina Maria Salvatore Freitas, Yalil Rodríguez

**Affiliations:** a Department of Health Sciences University of the Hemispheres, Quito, Ecuador; b Department of Orthodontics, Ingá University Center Uningá, Maringá, Paraná, Brazil

**Keywords:** Nickel–titanium archwires, orthodontics, toothpaste, toothbrushing, surface roughness

## Abstract

**Objective:**

The main objective of this *in vitro* study was to determine the effect of toothbrushing with fluoride and natural fluoride-free toothpastes on surface roughness changes of nickel-titanium (NiTi) wires as an indirect indicator of corrosion-related surface degradation.

**Materials and Methods:**

Thirty rectangular superelastic NiTi wires (0.019 × 0.025”, 20 mm length; Orthometric) were randomly assigned to three groups (*n* = 10 each): Group 1 – fluoride toothpaste with brushing; Group 2 – fluoride toothpaste without brushing; Group 3 – fluoride-free toothpaste with brushing. Brushing was simulated with an electric toothbrush twice daily for 1 min over 28 days (cumulative 1 h). Initial and final surface roughness (Rz, µm) was measured using a portable roughness tester. Data were analyzed using paired Student’s t-test and ANOVA with Tukey post hoc comparisons (*p* < 0.05).

**Results:**

Significant increases in surface roughness were observed in Group 1 (0.717 ± 0.085 to 2.440 ± 0.225 µm; *p* < 0.001) and Group 2 (0.791 ± 0.082 to 1.904 ± 0.247 µm; *p* < 0.001). In contrast, Group 3 showed only a slight and non-significant increase in surface roughness (*p* = 0.167). Intergroup analysis revealed significant post-treatment differences (*p* < 0.001), with Tukey’s test distinguishing three subsets: Group 1 > Group 2 > Group 3.

**Conclusions:**

Fluoride-containing toothpaste, particularly when combined with brushing, significantly increased surface roughness of NiTi wires, likely due to fluoride-induced degradation of the protective titanium oxide layer, which increases susceptibility to surface deterioration. Clinically, these findings suggest that fluoride-free formulations may represent a safer alternative for orthodontic patients requiring prolonged use of NiTi wires.

## Introduction

Corrosion of orthodontic archwires, particularly those made of nickel-titanium (NiTi), is a notable clinical concern given their constant exposure to the oral environment, which features fluctuations in salivary pH, temperature variations, biofilm presence, and chemical agents introduced through oral hygiene practices [1, 2]. Notably, fluoride-containing toothpastes, commonly recommended for caries prevention, have been implicated in compromising the integrity of these metallic materials. Such degradation may result in deterioration of superelastic properties, increased brittleness, and, in some cases, fracture of the wires, compromising treatment efficacy and posing potential risks to patient health through ion release and altered oral acidity [3, 4].

As an alternative, natural-origin non-fluoridated toothpastes have gained attention for their potential benefits: absence of synthetic dyes, lower ingestion-related toxicity, improved ecological footprint, and reduced risk of fluorosis in pediatric populations [5]. Meanwhile, toothbrushing technique introduces considerable variability in its impact, from patient dexterity and brushing duration to frequency [6], factors that are exacerbated during orthodontic treatment, with the use of specialized brushes and increased brushing periods.

A treatment involving fixed appliances demands hygiene tools that preserve biomechanical performance, avoiding interference with the loading cycles or structural stability of NiTi archwires. This prompts the key research question: What is the effect of toothbrushing with fluoride versus natural-origin non-fluoridated toothpastes on corrosion-related surface changes of NiTi orthodontic wires *in vitro*? Addressing this gap, the present study aims to inform clinicians about selecting oral hygiene products that are compatible with ongoing orthodontic treatment, minimizing adverse material interactions and systemic risk.

NiTi alloys, prized in orthodontics for their superelasticity, shape memory, and flexibility, are predominantly used in the early alignment phase of treatment [7, 8]. These wires must balance critical mechanical properties, fracture resistance, resilience, malleability, corrosion resistance, and biocompatibility for prolonged in-mouth use [9]. While other archwire materials like stainless steel, cobalt-chrome, and beta-titanium are used in various treatment phases [10], NiTi remains unique due to its mechanical characteristics.

A passive oxide layer, mainly titanium oxide, provides NiTi with corrosion and fracture resistance [11]. However, fluoride ions in oral hygiene products can degrade this protective layer, leading to electrochemical corrosion, increased friction in brackets, and potential systemic release of nickel ions [12]. Corrosion is linked to the acidic environment and fluoride-mediated breakdown of passive films [13, 14]. Additionally, wire thickness influences corrosion: thinner NiTi wires (e.g. 0.014”) corrode more aggressively under varying pH conditions than thicker ones [15].

Brushing mechanics, brush type, pressure, speed, and orientation, can cause surface wear and impact both enamel and orthodontic materials [16, 17]. Although electric brushes are suggested to reduce abrasiveness and benefit hygiene in orthodontic patients [18], biofilm accumulation remains a concern, with mixed evidence comparing electric and manual brushes in fixed appliance users [19].

Prior studies have demonstrated that fluoride-consuming products accelerate corrosion of orthodontic alloys, weakening structural integrity and promoting nickel ion release [3, 20–22]. For instance, NiTi wires exhibit higher surface roughness and reduced corrosion resistance when exposed to acidulated phosphate fluoride or high fluoride concentrations [20, 22], while fluoride-containing mouthwashes similarly elevate ion release [23]. Corrosion-induced fractures in NiTi wires have also been observed in fluoride solutions, but were not seen in neutral salt controls [24].

In contrast, there is a scarcity of studies assessing the effect of natural-origin non-fluoridated toothpastes on NiTi wire corrosion-related surface changes. To address this gap, the present study investigates, *in vitro*, the effect of toothbrushing with fluoride and natural non-fluoridated toothpastes on NiTi wire corrosion-related surface changes. The null hypothesis is that brushing with natural non-fluoridated toothpaste yields corrosion-related surface changes similar to those caused by fluoride-containing toothpaste. Findings will guide evidence-based selection of oral hygiene products to preserve NiTi wire integrity and optimize orthodontic treatment outcomes.

## Material and methods

### Study design

This experimental *in vitro* study aimed to evaluate the effect of toothbrushing with fluoride and non-fluoride toothpastes on the surface roughness of superelastic NiTi wires as an indirect indicator of corrosion-related degradation. The sample consisted of 30 rectangular NiTi wires (Orthometric, Brazil), each measuring 0.019 × 0.025 inches and 20 mm in length. For each wire, the initial and final surface roughness were assessed.

### Experimental groups

The wires were randomly allocated into three groups (*n* = 10 per group):

- Group 1 (Fluoride + Brushing): NiTi wires brushed with a fluoride toothpaste.
- Group 2 (Fluoride only): NiTi wires exposed to fluoride toothpaste without brushing.
- Group 3 (Non-fluoride + Brushing): NiTi wires brushed with a natural non-fluoride toothpaste.

### Brushing simulation

Brushing was simulated twice daily for 1 min over 28 days, corresponding to a total of 60 min of exposure (1 h), using an electric toothbrush (Oral-B iO10, Procter & Gamble, USA). Authorization to conduct the study was obtained from the University of the Hemispheres, and measurements were performed in the Materials Laboratory under the supervision of an expert supervisor.

### Wire preparation and initial roughness

Each NiTi wire segment (20 mm) was measured with a Vernier caliper (Pretul, Truper S.A., Mexico) and cut with a distal end cutter. Initial surface roughness was measured using a portable roughness tester (MarSurf PS 10, Mahr, Germany), which records micrometer-scale deviations using a diamond stylus at a scanning speed of 0.5 mm/s. Three readings per wire were averaged to determine baseline roughness values.

### Saliva immersion

All wire segments were immersed for 24 h in 20 mL of artificial saliva (Saliv by Denture, Lamosan, Ecuador) contained in short glass vials. According to the manufacturer, the formulation includes water, sorbitol, carboxymethylcellulose, xylitol, disodium phosphate, sodium phosphate, potassium chloride, sodium chloride, calcium chloride, magnesium chloride, methylparaben, propylparaben, and saccharin sodium.

### Brushing protocol

After drying at room temperature for 30 min, each wire was stabilized in an acrylic base (3 × 3 × 1 cm) to simulate brushing. A standardized amount of 0.25 g of toothpaste was applied per protocol by Creeth et al. [25], using a digital balance (MagicTeck, China).

- Group 1: Fluoride toothpaste (Encident Brackets®, Blenastor, Spain; 1,450 ppm NaF, sorbitol, silica, glycerin, xylitol, potassium pyrophosphate, chamomile extract, panthenol, chlorhexidine digluconate 0.04%, zinc chloride 0.07%). Brushing was performed for 1 h (cumulative 28 days).
- Group 2: The same fluoride toothpaste was applied directly to the wire surface without brushing.
- Group 3: Non-fluoride natural toothpaste (Propolis Dental®, La Melífera, Ecuador; formulation includes water, beeswax, honey, aloe vera, lanolin, chamomile extract, propolis, and natural oils). Brushing followed the same protocol as Group 1.

### Post-treatment procedures

After brushing, wires were removed with cotton pliers, rinsed for 30 s in distilled water (Nova Laboratory, Brazil) to remove toothpaste residues, and air-dried on filter paper for 30 min. Final roughness was measured using the same roughness tester, and the Rz parameter (mean height of the five highest peaks and deepest valleys) was calculated.

### Statistical analysis

All data were recorded in Microsoft Excel (Microsoft Corp., USA) and analyzed with SPSS Statistics v27 (IBM, Armonk, NY, USA). Normality was tested using the Shapiro–Wilk test. As the data were normally distributed, parametric tests were performed: paired Student’s *t*-test for within-group comparisons (before vs. after) and one-way ANOVA for intergroup comparisons. A significance level of *p* < 0.05 was adopted.

## Results

The intragroup comparison is summarized in Table 1. In the group brushed with Encident toothpaste (Group 1), surface roughness increased markedly from a baseline mean of 0.717 µm (standard deviation [SD] 0.085) to 2.440 µm (SD: 0.225), with statistically significant differences confirmed by the paired Student’s *t*-test (*p* < 0.001). The group exposed to Encident toothpaste without brushing (Group 2) also showed a significant increase, rising from 0.791 µm (SD: 0.082) to 1.904 µm (SD: 0.247) (*p* < 0.001). In contrast, the group brushed with fluoride-free toothpaste (Group 3) exhibited only a slight, non-significant increase, from 0.723 µm (SD: 0.099) to 0.828 µm (SD: 0.230) (*p* = 0.167).

**Table 1:** Comparisons of initial and final surface roughness of each group.

Groups		*N*	Mean	SD	*p*
Encident toothpaste with brushing	Initial	10	0.717	0.085	0.000*
	Final	10	2.440	0.225	
Encident toothpaste without brushing	Initial	10	0.791	0.082	0.000*
	Final	10	1.904	0.247	
Fluoride-free toothpaste with brushing	Initial	10	0.723	0.099	0.167
	Final	10	0.828	0.230	

* Statistically significant for *p* < 0.05.

The intergroup comparison is shown in Table 2. At baseline, no significant differences were observed among the three groups (*p* = 0.139). After treatment, however, highly significant differences emerged (*p* < 0.001). Tukey’s post hoc test revealed three distinct subsets: the lowest roughness values were recorded in Group 3 (0.828 µm), followed by Group 2 (1.904 µm), while Group 1 displayed the highest values (2.440 µm).

**Table 2:** Intergroup comparison of initial and final surface roughness.

Groups		*N*	Mean	SD	*p*
Initial	Encident toothpaste with brushing	10	0.717	0.085	0.139
	Encident toothpaste without brushing	10	0.791	0.082	
	Fluoride-free toothpaste with brushing	10	0.723	0.099	
	Total	30	0.743	0.092	
Final	Encident toothpaste with brushing	10	2.440 A	0.225	0.000*
	Encident toothpaste without brushing	10	1.904 B	0.247	
	Fluoride-free toothpaste with brushing	10	0.828 C	0.230	
	Total	30	1.724	0.718	

* Statistically significant for *p* < 0.05.

Different letters in the same column indicate the presence of a statistically significant difference between the groups, as indicated by the Tukey test.

Overall, a fluoride-containing toothpaste (Encident) caused the greatest increase in surface roughness of NiTi wires, both with and without brushing, whereas brushing with a fluoride-free toothpaste produced minimal changes that were not statistically significant (Table 2).

## Discussion

The purpose of this study was to evaluate *in vitro* the effect of toothbrushing combined with fluoride and fluoride-free toothpastes on corrosion-related surface changes of nickel–titanium orthodontic archwires. Preventive use of fluoride-based products is widely recommended in orthodontic patients due to the increased risk of plaque accumulation and enamel demineralization around brackets [26]. However, fluoride ions have also been implicated in altering the corrosion resistance of NiTi alloys, potentially compromising their mechanical and clinical performance [3, 7].

Surface roughness was used as an indirect indicator of corrosion-related surface degradation; however, no direct electrochemical corrosion analysis was performed. Our results demonstrated that fluoride-containing toothpaste, particularly when combined with brushing, significantly increased surface roughness of NiTi wires compared with baseline and fluoride-free toothpaste, likely due to fluoride-induced disruption of the protective titanium oxide layer, which increases susceptibility to surface degradation. This agrees with previous *in vitro* and *in vivo* findings showing that fluoride exposure accelerates electrochemical corrosion of NiTi wires, underscoring the direct chemical effect of fluoride on NiTi alloys through alteration of the protective oxide layer, resulting in loss of elasticity, higher frictional resistance, and increased risk of fracture [13, 27]. Belasic et al. [7] further confirmed that NiTi alloys display good anticorrosive behavior in the absence of fluoride but develop significant deterioration at higher fluoride concentrations due to surface oxide disruption.

Notably, brushing with fluoride-free toothpaste caused only minimal and non-significant changes in surface roughness, indicating that natural fluoride-free dentifrices may provide a safer alternative during orthodontic therapy. This supports earlier reports suggesting that casein phosphopeptide–amorphous calcium phosphate (CPP-ACP) formulations may offer anticariogenic benefits without promoting corrosion of metallic appliances [28].

Our findings also highlight that fluoride exposure, even without brushing, increased surface roughness more than brushing with fluoride-free toothpaste, underscoring the direct chemical impact of fluoride on NiTi alloys. Similar results were reported by Abbassy [21] and Kassab and Gomes [24], who found that NiTi wires exposed to fluoride-containing solutions exhibited surface degradation and higher nickel ion release compared to control conditions.

The influence of brushing itself should not be underestimated. While most previous studies have simulated brushing primarily to assess enamel or denture material abrasion [16, 19], our results demonstrate that brushing may exacerbate surface changes when combined with fluoride dentifrices, possibly due to synergistic tribocorrosion effects [5]. This finding adds new evidence to the literature, as few studies have experimentally addressed brushing plus toothpaste composition on orthodontic alloys.

Clinically, the degradation of NiTi wires may result in decreased superelasticity, loss of force constancy, and premature replacement, thereby prolonging treatment time and increasing costs [8, 11]. More importantly, ion release from corroded wires raises biocompatibility concerns, since nickel is a well-recognized allergen and may elicit systemic effects [12, 29]. Therefore, careful consideration of oral hygiene recommendations in orthodontic patients is essential. While fluoride remains fundamental for caries prevention [30], clinicians should balance its benefits with the potential risk of accelerated corrosion of NiTi appliances. Alternatives such as fluoride-free or CPP-ACP toothpastes may represent valid adjuncts, particularly in patients with high sensitivity to nickel or those requiring prolonged use of NiTi wires.

## Limitations and future directions

This study was limited by its *in vitro* design, single brand of NiTi wires, and sample size. The brushing simulation, although standardized, cannot fully replicate intraoral conditions, such as variable pH, temperature fluctuations, and dietary factors [6]. Future *in vivo* studies are warranted to validate these findings under clinical conditions and to explore the long-term effects of different oral hygiene regimens on orthodontic wires.

## Conclusions

The null hypothesis was rejected, as fluoride-containing toothpastes combined with brushing produced a significantly greater increase in the surface roughness of NiTi archwires compared to a natural fluoride-free formulation. The lowest surface alteration was observed with fluoride-free toothpaste combined with brushing, indicating reduced surface degradation under these conditions. Importantly, even in the absence of brushing, fluoride-containing toothpaste increased surface roughness, likely due to disruption of the protective oxide layer, highlighting the intrinsic effect of fluoride ions on NiTi surface characteristics.

## Data Availability

The data generated and analyzed during the current study are available from the corresponding author on reasonable request.
